# The Effect of Whey and Soy Protein Isolates on Cognitive Function in Older Australians with Low Vitamin B_12_: A Randomised Controlled Crossover Trial

**DOI:** 10.3390/nu11010019

**Published:** 2018-12-21

**Authors:** Ian T. Zajac, Danielle Herreen, Kathryn Bastiaans, Varinderpal S. Dhillon, Michael Fenech

**Affiliations:** Health & Biosecurity, Commonwealth Scientific and Industrial Research Organisation (CSIRO), 5000 Adelaide, South Australia, Australia

**Keywords:** whey protein isolate, dairy, cognitive function, cobalamin, vitamin B_12_

## Abstract

Whey protein isolate (WPI) is high in vitamin B_12_ and folate. These and other related markers (holotranscobalamin, methylmalonic acid and homocysteine) have been linked with cognitive health. This study explored the efficacy of WPI for improving cognitive function via delivery of vitamin B_12_. Moderately vitamin B_12_-deficient participants aged between 45 and 75 years (*n* = 56) were recruited into this randomised controlled crossover trial. Participants (55% female) consumed 50 g whey (WPI; active) or soy protein isolate (SPI; control) for eight weeks. Following a 16-week washout phase, they consumed the alternative supplement. Consumption of WPI significantly improved active B_12_ and folate status but did not result in direct improvements in cognitive function. However, there was evidence of improvement in reaction time (*p* = 0.02) and reasoning speed (*p* = 0.04) in the SPI condition for females. Additional analyses showed that changes in active B_12_, HcY and folate measures during WPI treatment correlated with improvements in cognitive function (all *p* < 0.05). Results indicate that WPI itself did not result in improved cognitive function but some evidence of benefit of SPI for females was found. However, consistent with previous research, we present further evidence of a role for active B_12_, HcY and folate in supporting cognitive improvement in adults with low B vitamin status.

## 1. Introduction

The impact of cognitive decline in a dramatically ageing population is one of the biggest challenges of the future, given its impact on both the individual and society [1]. Not only is deterioration of cognitive functioning amongst the most feared aspects of growing old due to its ability to lower a person’s quality of life, it is often accompanied by significant burden [2]. Accordingly, there is a growing need to identify practical methods of delaying cognitive decline to ensure independence in aged individuals. 

Epidemiological and observational studies suggest a relationship exists between nutrient deficiencies and cognitive decline in ageing adults [3,4,5]. In the case of vitamin B_12_, low levels (together with low folate levels) are a common cause of elevated total homocysteine (HcY), which has been shown to be an independent risk factor for cognitive decline and Alzheimer’s disease [6,7,8,9,10,11,12]. Vitamin B_12_ deficiency is common in older adults, with prevalence increasing substantially with age [13,14,15,16,17,18]. In the UK, three surveys reported that around 1 in 20 people aged 65–74 years and at least 1 in 10 of those aged ≥75 years were deficient in vitamin B_12_ [18]. Moreover, an Australian survey of persons aged ≥50 years reported 23% of participants to have low vitamin B_12_ levels, with prevalence increasing to ~30% for men aged ≥70 years and women ≥80 years [13]. 

Over the past few decades, studies have produced inconsistent findings regarding vitamin B_12_ and its association with cognitive impairment in older adults [12]. A recent review of prospective cohort studies [19] found no association between serum vitamin B_12_ concentrations and cognitive decline. However, it was noted that the four studies that used alternate biomarkers of vitamin B_12_, that is, methylmalonic acid (MMA; a marker of progressed vitamin B_12_ deficiency) and holotranscobalamin (the biologically active fraction of this vitamin and hereafter referred to as active B_12_) did find evidence of an association with cognitive status [20,21,22,23]. Moreover, these markers of vitamin B_12_ deficiency appear to display a stronger relationship with cognition than serum B_12_ [16,24,25,26]. 

A 10-year longitudinal cohort study of 1648 individuals [20] found that a doubling of MMA was associated with a 50% faster rate of cognitive decline, with a doubling of active B_12_ associated with a 30% slower rate of decline in individuals aged over 65 years. Furthermore, a 5-year prospective study of 107 community-dwelling participants aged 61–87 years without cognitive impairment at enrolment showed that the decrease in brain volume was greatest among those with lower serum vitamin B_12_ and active B_12_ levels and higher plasma total homocysteine (HcY) and MMA levels at baseline [27]. These results are consistent with findings by Tangney et al. [28], in which high HcY and MMA levels were associated with rate of cognitive decline, along with a decrease in total brain volume.

A recent comprehensive review of the literature on the association of dairy foods and cognitive function in older adults concluded that “low-fat dairy products, when consumed regularly as part of a balanced diet, may have a number of beneficial outcomes for neuro-cognitive health during ageing” and identified the whey fraction as being particularly rich in a range of bioactives, including proline-rich polypeptides, α-lactalbumin and B_12_, which may support brain health in diverse ways [10]. Whey is a natural by-product of the manufacture of cheese, with 100 g of dried powder providing 2.5 µg of vitamin B_12_, equivalent to 100% of the Recommended Dietary Allowance (RDA). Acid whey powder contains high amounts of lactose (60–70 g per 100 g), which is problematic given the increased risk of lactose malabsorption in older adults [29]. Whey protein isolate (WPI), on the other hand, has less than 1 g lactose per 100 g and is also richer in vitamin B_12_ (6 µg/100 g). Additionally, WPI has an 80% lower level of sodium and 14-fold higher level of natural folate relative to acid whey powder. While WPI has already been shown to be beneficial for improving cognitive performance in the context of memory tasks in stress-vulnerable subjects [30], the bioavailability and bioefficacy of vitamin B_12_ from WPI remains relatively unexplored, particularly in relation to age-related cognitive decline. 

The purpose of the present study was to consider the role that dairy-based products such as WPI may have on nutritional markers associated with age-related cognitive decline. Primary outcomes including the impact of WPI supplementation on vitamin B_12_ status and related factors in individuals with subclinical B_12_ levels at baseline have already been reported [31] but are briefly revisited herein. This study specifically explores the impact of WPI supplementation on cognitive function measured during the intervention. Furthermore, it explores the relationship between changes in various biomarkers of B_12_ status (serum B_12,_ active B_12_, MMA and HcY) and changes in cognitive function during the course of the active intervention.

## 2. Materials and Methods

### 2.1. Screening and Recruitment of Participants

**Figure 1:**
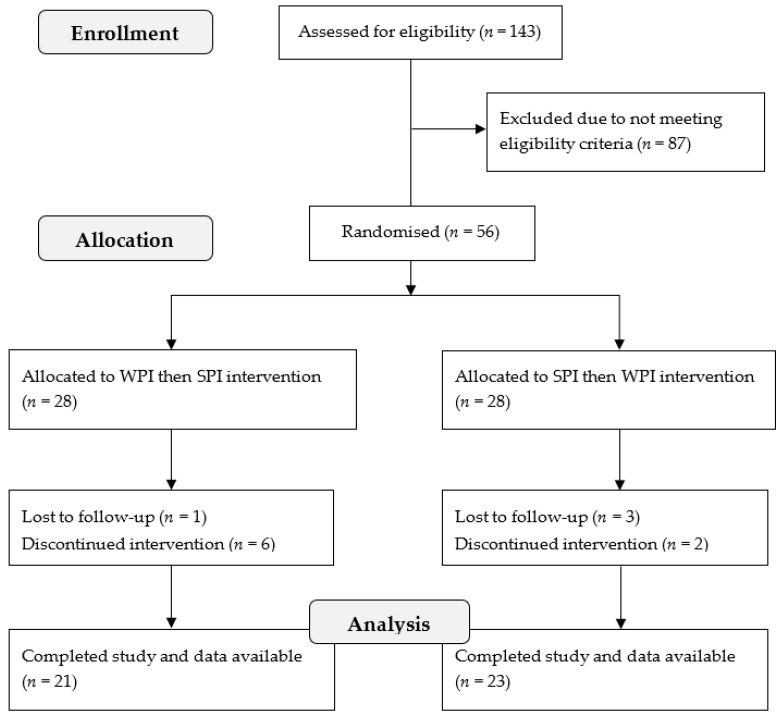
Consort diagram of the intervention trial.

The study was advertised in local newspapers in the Adelaide metropolitan area and on the CSIRO clinic website. Interested participants contacted the clinic to arrange an appointment for screening. Fifty-six eligible participants attended an information session and read the study information sheet before providing written informed consent to participate in the study. Figure 1 provides an overview of screening and recruitment and Table 1 provides an overview of baseline characteristics of study completers. All data collection visits for this study occurred between September 2014 and June 2015.

Inclusion criteria were: healthy subjects aged between 45 and 75 years, not taking vitamin B_12_/choline/antioxidant vitamins at doses that exceed 25% of the Recommended Dietary Allowance, subclinical vitamin B_12_ deficiency as defined previously [31] (using the following parameters: serum concentration of B_12_ in the range of 100–350 pmol/L, plasma MMA >0.20 µmol/L and serum creatinine concentration of 120 µmol/L or less) and willing to consume the quantities of WPI or SPI specified for the trial.

Exclusion criteria were: cognitive deficiencies indicated by Mini Mental State Examination Score ≤24, current smokers, people who habitually consume more than two standard alcoholic drinks per day, BMI ≥35 kg/m^2^, diagnosed with diabetes and/or lactose intolerance, history of pernicious anaemia or atrophic gastritis and regular users of antacids. 

### 2.2. Intervention Design

This study was a registered (ACTRN12614000159651) randomised controlled crossover intervention, which was approved by the CSIRO Human Research Ethics Committee. Participants were randomised to daily intake of 50 g WPI or 50 g SPI (control) for eight weeks. Following an intervening 16-week washout phase, participants crossed over to the alternative supplement for the final eight weeks. The randomisation scheme was generated by the clinical trials manager using the online resource website: http://www.randomization.com. Both products were packaged in matching opaque containers with a numeric allocation number to ensure blinding of participants, researchers and clinical trial staff.

Participants were asked to maintain their habitual diet during the intervention trial but refrain from eating foods high in vitamin B_12_ (i.e., foods with vitamin B_12_ levels > 4.9 µg/100 g) and also refrain from taking supplements containing vitamin B_12_/choline/antioxidants). Dietary restrictions included limiting consumption of vegetables or pulses (2 servings/day), fruits or juices (3 servings/day), black tea or coffee (2 cups/day), chocolate (50 g/day), wine (200 mL/day) and/or beer (375 mL/day) in order to avoid an excessive intake of antioxidants to minimise variation between subjects and groups. Participants completed 3-day food and beverage intake records at baseline and endpoint to monitor their adherence to these restrictions and were supported throughout the study by a dietitian. 

The WPI or SPI was consumed daily as 25 g blended in 200 mL fruit juice or water twice a day so that the total daily intake was 50 g WPI or 50 g SPI each day. The WPI and SPI were provided in 25 g sachets (two per participant for each day of the intervention phases). A compliance checklist for use of the WPI and SPI sachets was completed by the participants. 

During the washout period, participants were allowed to return to their habitual dietary habits and were required to avoid consumption of supplements containing vitamin B_12_ or foods fortified with vitamin B_12_. Serum B_12_ was checked mid-way during the washout period. Those exceeding 350 pmol/L serum B_12_ concentration were required to restrict intake of foods rich in B_12_ until they achieved a < 350 pmol/L serum concentration before starting the second treatment phase. 

### 2.3. Nutritional Profile of WPI and SPI

The nutritional composition of WPI and SPI is shown in Table 2. According to the manufacturer’s product data, 100 g of WPI and SPI contributed 6 and 0 µg of vitamin B_12_, respectively. Independent analysis by PathWest Laboratory Medicine WA (Nedlands, Western Australia, Australia) showed that WPI contained 5.64 µg of vitamin B_12_ per 100 g, whereas the amount of vitamin B_12_ in SPI was less than 0.34 µg/100 g. 

### 2.4. Biochemical Outcome Measures

Blood samples were collected at the beginning and end of each of the intervention phases and half-way through the washout phase, that is, at 0, 8, 16, 24 and 32 weeks, within the clinical research unit. Blood samples were used fresh or biobanked frozen at −80 °C depending on the requirements of each assay. The biochemical outcome measures included in the present analyses were: serum B_12_, active B_12_ (holotranscobalamin), methylmalonic acid (MMA), plasma total homocysteine levels (HcY) and serum folate. Complete descriptions of these measures and associated laboratory processing and results have been described elsewhere [31].

### 2.5. Tests of Cognitive Function

The CSIRO Cognitive Assessment Battery (C-CAB) comprised of 10 individual cognitive tasks that measure cognitive domains including processing speed, reaction time/attention, reasoning speed, verbal and numeric working memory, immediate and delayed word memory. The tasks utilised obtain latency/response time and accuracy data with performance outcomes for each measure reflecting both of these components. The test battery was programmed to run in the Inquisit version 4 environment [32] and the battery took approximately 45 minutes to complete. Participants completed the C-CAB at each of their clinic visits. The tests used to measure performance on each of the cognitive domains are now described in the order completed.

#### 2.5.1. Immediate Word Memory

Participants were presented with a list of 15 words obtained from the MRC Psycholinguistic Database [33], which included one-to-two syllable words with high concreteness (Mean = 572.81, SD = 35.92) and meaningfulness (Mean = 681.87, SD = 83.31) ratings. Prior to presentation, participants were instructed to remember the words and were informed they will be tested immediately, as well as after approximately 20 minutes. Words were presented in the middle of the computer, one at a time, with a display time of 2-seconds and an inter-stimulus interval (ISI) of 500-millseconds (msec). Parallel word lists were used for alternate testing sessions to remove practice effects. Immediately after presentation, participants were tested using a 30 words list. The list included the original 15 words plus 15 new distractors. Words were shown one-at-a-time in the centre of the computer screen with a 500 msec ISI between the participant’s response and subsequent word stimulus. Participants indicated whether the word was from the initial word list or not, by pressing ‘Yes’ and ‘No’ response keys. Raw outcome measures included mean response latency—time between stimulus onset and participant’s response—and accuracy measures. 

#### 2.5.2. Processing Speed

The first task assessing processing speed was adapted from previous research [34]. A code table was presented at the top of the computer screen and comprised nine symbols arranged horizontally, to which nine digits, presented directly beneath them, were paired. For each item, one symbol was presented in the centre of the computer screen and participants responded by left clicking the mouse on its corresponding digit in a 3 × 3 numerical response grid positioned at the bottom of the screen. The raw outcome measure was the number of items correctly completed in 90 s.

The second task was a computerised string search task adapted from the ETS Kit of Factor Referenced Tests [35]. In this task, participants were required to decide whether two number strings, presented side-by-side, were identical and press the ‘Yes’ or ‘No’ key on the keyboard accordingly. The raw outcome measure was the number of items completed correctly in 90 s. 

#### 2.5.3. Reaction Time/Attention

Two-choice and four-choice reaction time tasks were used to measure this domain and were adapted from our previous studies [34]. To begin each trial, the participant pressed the ‘H’ key on a response keypad. This action triggered a cue symbol (+) of approximately 40 px which appeared in the centre of the computer screen together with two or four black bordered empty target squares (100 px) positioned 150 px each side of the cue (left and right for two-choice, with addition of top and bottom square for four-choice). After a variable ISI ranging from 1000 msec to 2500 msec, a black arrow was presented in one of the squares (arrow direction was congruent with location; pointing left, right, up or down). Participants responded by pressing the corresponding response arrow in the response keypad as quickly as possible (response keys were positioned left, right, above and below the ‘H’ key). Practice phases required participants to complete four and eight trials correctly. Mean response latencies for each task were subsequently estimated over 30 valid trials defined as: 1) response was correct; 2) response latency was not less than 150 ms; and 3) response latency was not greater than mean latency + 3.5 times the intra-individual standard deviation of latencies obtained during the second phase of practice trials in each task.

#### 2.5.4. Verbal Working Memory

This was measured using a letter memory task adapted from previous studies [36]. In this task, participants were initially instructed to memorise a set of five letters presented sequentially on the computer screen for 2000 msec each with an ISI of 500 msec. Following a warning probe of 3000 msec, 31 test trials commenced and participants were required to indicate whether the test letter displayed was in the original learned set or not. In the second phase of this task, participants were required to remember a new set of eight letters which did not include the original five, followed by 31 test trials as per above. Raw outcome measures included mean response latency and accuracy.

#### 2.5.5. Numerical Working Memory

This was measured using a number memory task adapted from previous studies [36] and was operationally equivalent to the verbal working memory task described above. However, in this task, participants were instructed to memorise sets of numbers (either five or eight) and to make yes/no decisions about numbers presented subsequently in the test phase. Raw outcome measures included mean response latency and accuracy.

#### 2.5.6. Delayed Word Memory

Approximately 20 minutes into the cognitive battery, participants were tested again on the 15 words presented at the start of the session. The test consisted of 30 trials involving the original 15 words, plus 15 new distracters not used in the immediate recognition test. All other presentation parameters and scoring parameters were as per the immediate recognition test described above. 

#### 2.5.7. Reasoning Speed

Two tasks adapted from the odd-man-out reaction time paradigm were used to assess this domain [37]. The tests used are more complex than simple reaction time because they assess the ability to make reasoned decisions concerning distal relationships between stimuli [38]. In the first version, trials consisted of eight circles arranged horizontally with the total stimulus set being 640 px wide and 84 px high. In each set, three target circles were illuminated red and two targets were closer together in relation to the third target. Participants indicated to which side-left or right-of the two closer targets the odd-man-out (i.e., third target) was located. Participants completed two practice phases consisting of six trials each. The test-phase consisted of 40 trials and raw outcome measures included mean response latency and accuracy.

The second task is procedurally equivalent to the first task. However, in this version, trials consisted of eight mixed shapes (squares, diamonds and triangles) arranged horizontally. The target stimuli were triangles as opposed to illuminated circles and participants indicated to which side—left or right—of the two closer triangles the odd-man-out (i.e., third triangle) is located. Outcomes measures were as per above.

### 2.6. Sample Size, Data Processing and Analysis

The required sample size for this study was estimated using GPower. Results showed a minimum of *n* = 74 participants were required for a repeated-measures model assuming a small within-between interaction effect (η^2^ = 0.02) with 90% power. For crossover designs, as used herein, this figure is halved, requiring a minimum of *n* = 37 participants. The number of recruited participants who completed this study was *n* = 44, thus satisfying statistical power requirements.

Transformations were undertaken to normalise distributions of cognitive tasks for analysis purposes. In line with previous studies [39], work rates representing the number of items completed per second were calculated. Then, these work rates were adjusted for accuracy (work rate *×* % correct). Finally, performance was averaged across tasks measuring the same domain (e.g., reaction time/attention) to arrive at a composite measure.

Cognitive data were analysed at the Intention-to-Treat level and therefore included all participants who commenced the trial (whether or not they withdrew). Change in cognitive function was assessed using linear mixed effects models—capable of handling missing data—and the repeated nature of the measurements were accounted for by permitting random slopes and intercepts for each participant for each treatment. Fixed effects included time, treatment and time by treatment interactions. An unstructured covariance matrix was specified for random effects and all models controlled for participant’s sex, age and the order of assigned treatments. The assumption of normally distributed residuals was assessed and was satisfied for all models.

The final analyses assessed the relationship between changes in vitamin B_12_ and related parameters occurring during the WPI intervention period only and corresponding changes in cognitive function using first-order correlations adjusted for sex and age covariates. The calculation of change scores was thus based only on those who attended final follow-up (*n* = 44) and who therefore had complete data.

## 3. Results

Participants in this sample had a mean age of 60 years (SD = 8.5 years) and were reasonably well balanced in regard to sex (55% female). Table 3 provides average levels of cognitive performance over the course of the study as well as mean change in cognitive function for each domain. Equivalence of the groups at baseline was confirmed via no statistically significant differences between treatment arms for any measure (all *p* ≥ 0.42). Descriptive statistics regarding changes in cognition show that it was generally neutral or positive.

**Figure 2:**
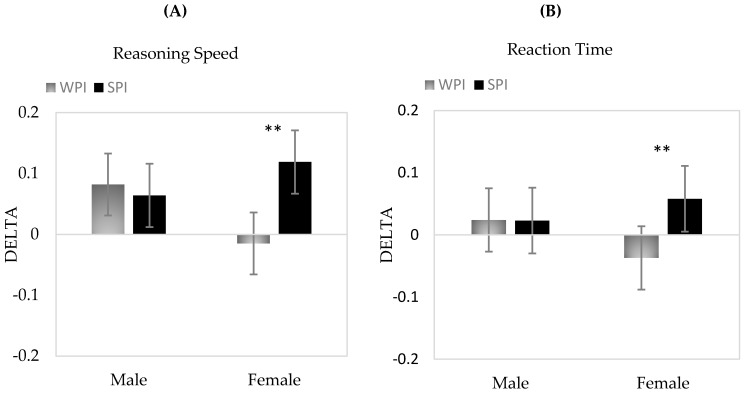
Mean change scores and 95% Confidence Intervals for Treatment × Gender interactions for Delta (**A**) Reasoning Speed and (**B**) Reaction Time. **p < 0.01.

Analysis of data using mixed effects models showed significant overall time effects for reasoning speed, verbal working memory and numeric working memory (all *p* ≤ 0.001). In addition, there were significant time by treatment interaction effects observed for reasoning speed (*p* = 0.04) and reaction time (*p* = 0.02). In both cases, performance increased over the duration of the intervention with a significantly greater increase observed during the SPI compared to WPI treatment. Additional three way interactions (time by treatment by sex) showed that these effects were only evident in females during SPI treatment (both *p* < 0.05). As shown in Figure 2, there was generally no change in performance for males during either treatment for either cognitive domain but significant direct changes were observed for females during SPI treatment only.

The results of this intervention on various biochemical scores have been reported in full previously [31]. However, given the presence of prior studies suggesting a relationship between alternate markers of B_12_ status and cognition, additional analyses were conducted in order to examine changes in markers of B_12_ status during the active WPI treatment phase only and their associations with observed changes in cognitive function during the same treatment period. Briefly, the results of the intervention on the parameters of interest are provided in Table 4. WPI treatment lead to significant direct changes in active B_12_ and serum folate, however changes in MMA did not quite reach significance. Correlations between changes in these biochemical measures and cognitive function during both WPI and SPI intervention periods can be seen in Table 5. These correlations were fully adjusted for age and sex covariates. As can be seen, 5/6 significant correlations emerged for the WPI intervention. Increases in in active B_12_ were associated with improvements in processing speed. Similarly, increased serum folate was associated with improvements in delayed word memory. Reductions (i.e., improvements) in homocysteine levels were related to improvements in processing speed, numeric working memory and verbal working memory. In the SPI condition, homocysteine changes were related to reasoning speed with higher levels equating to reduced performance. We conducted the same analyses using combined B_12_ (cB_12_), a combined indicator of corrected B_12_ status [31] and the pattern of correlations was the same as for active B_12_.

## 4. Discussion

This study sought to examine the effects of WPI and SPI on cognitive function and vitamin B_12_ status in low B_12_ individuals. Cognitive function was measured for a variety of important domains using a robust cognitive assessment battery. Overall, there were no effects of WPI on cognitive function, which generally remained stable across visits during this treatment. On the contrary, SPI appeared to have benefits for the cognitive domains of reasoning speed and reaction time in females only. Given the age of participants in this sample (45–75 years) and the presence of this effect true for females only, this finding possibly reflects the impact of soy isoflavones on cognitive function in post-menopausal women [40]. A recent meta-analysis concluded that soy isoflavones exert a positive effect on a broad range of cognitive abilities in females and that this effect is robust in women as young as 60 years [41]. 

Despite the absence of a main treatment effect for WPI in terms of its ability to improve cognitive performance, positive findings were evident regarding changes in biometric measures of vitamin B_12_ status during WPI treatment and changes in cognitive function. Specifically, increases in active B_12_ and serum folate were associated with corresponding improvements in some cognitive domains. Reductions in homocysteine levels were also consistently related to improvements in cognitive performance, including working memory. Whilst there were no clear treatment effects linking WPI specifically to improved cognitive function, the changes observed in these key biochemical markers and the relation of these to improved cognition were consistent with the broader research literature regarding the role of B_12_, folate and homocysteine in helping to maintain good cognitive function [12]. 

The finding of direct associations between changes in biochemical and cognitive measures despite the absence of any effect directly attributable to WPI likely reflects the choice of the control treatment. More specifically, although SPI is a useful comparison given its different nutritional profile to WPI in terms of levels of B-vitamins and folate, the isoflavones present can have an impact on cognitive function, most notably in females [40]. Therefore, both treatments were arguably ‘active’ in relation to their ability to affect cognitive performance and the absence of any interaction effect favouring WPI does not preclude potential benefits of this for improving cognition as a result of its demonstrated influence on vitamin B-status. Thus, future studies will need to consider their choice of control treatment more carefully if the primary outcome concerns cognitive function. 

In addition to using a relatively active control treatment, this study employed a relatively short intervention duration (eight weeks). Prior research has proposed that interventions involving otherwise healthy participants, as used herein, require durations as long as 2-to-5 years in order to demonstrate an impact on cognitive function [42]. Thus, it has been suggested that such studies might benefit from recruiting individuals at-risk of enhanced rates of cognitive decline. Given the relationships observed herein between changes in B_12_ related markers and improved cognitive performance, future studies should consider the potential for dairy-based supplements to slow the rate of decline in individuals with quantifiable Age-Associated Memory Impairment (AAMI). These individuals are particularly interesting given the absence of underlying pathology (e.g., dementia) explaining their decline and the fact that they do not yet meet criteria for MCI [43].

The potential utility of using WPI or SPI for extended periods of time as a means of supplementing diets must be balanced against any potential negative impacts of these. For example, dairy (WPI) has been linked with self-reported gut discomfort and subsequent avoidance of such products [44]. Question marks also remain in relation to soy and its potential impact on endocrinology [45], as well as the presence of contaminants such as aluminium in soy products [46]. 

In conclusion, although this intervention did not demonstrate a direct effect of WPI supplementation on cognitive function, the results provide some support to the notion of vitamin B_12_ and folate status having a role in supporting cognitive health. Specifically, important vitamin B_12_ and folate related markers were improved as a result of WPI supplementation and these changes were correlated with improvements in cognitive performance. However, the findings from this study are limited to similarly aged adults with subclinical vitamin B_12_ deficiency and further research is needed to explore how these findings may relate to other groups and populations. 

## Figures and Tables

**Table 1:** Baseline characteristics of participants who completed the study.

Variable	Treatment Order	
	SPI then WPI (*n* = 23)	WPI then SPI (*n* = 21)
% Female (*n*)	43% (10)	52% (11)
Mean Age in years (SD)	61.8 (1.94)	61.1 (1.78)
Mean Body Mass Index (SD)	26.8 (0.76)	26.41 (0.88)

SPI: soy protein isolate; WPI: whey protein isolate.

**Table 2:** Nutritional composition of WPI and SPI as per manufacturer’s product specifications.

Composition	Type	Amount in WPI	Amount in SPI
Protein (%)	Total proteins	93.1 (%) Dry basis	91.0% Dry basis
	β-Lactoglobulin	45%	-
	α-Lactabumin	15%	-
	GMP (glycomacropeptide)	16%	-
	Minor components	3–5%	-
	Immunoglobulins	4%	-
	Bovine serum albumin	1%	-
	Lactoferrin	0.1%	-
Fat (%)	Total fats	1%	2.5%
	Saturated fats	-	0.8%
	Polyunsaturated	-	1.6%
	Monounsaturated	-	0.6%
	Trans fatty acids	-	0.5%
Carbohydrate (%)	Total carbohydrates	1.2%	1%
	Lactose	1.2%	-
	Sucrose	-	<1%
Vitamins (per 100 g)	Pantothenic acid	<1.0 mg	-
	Riboflavin (B_2_)	0.32 mg	-
	B_6_	0.22 mg	0.1 mg
	Thiamine (B_1_)	0.12 mg	-
	α-Tocopherol	<0.1 mg	-
	Niacin (B_3_)	<0.01 mg	-
	Folacin	458 µg	-
	B_12_	6.0 µg	0.0 µg
Minerals (per 100 g)	Potassium	1005 mg	1300 mg
	Calcium	307 mg	-
	Phosphorus	210 mg	-
	Sodium	188 mg	800 mg
	Chloride	11 mg	-
Polyphenols	Isoflavones	-	10–30 mg

**Table 3:** Means (± standard error of the mean) for cognitive variables at baseline, endpoint and for overall change.

Cognitive Domain	Treatment	Baseline		Endpoint		Change	
		Mean	± SE	Mean	± SE	Mean	± SE
Processing Speed	WPI	0.36	0.01	0.37	0.01	0.01	0.00
	SPI	0.37	0.01	0.37	0.01	0.00	0.00
Reasoning Speed	WPI	0.83	0.04	0.86	0.04	0.04	0.02
	SPI	0.79	0.04	0.88	0.04	0.09	0.02
Reaction Time/Attention	WPI	1.67	0.05	1.67	0.05	−0.01	0.02
	SPI	1.66	0.05	1.70	0.05	0.04	0.02
Numeric Working Memory	WPI	1.14	0.04	1.22	0.04	0.07	0.03
	SPI	1.18	0.05	1.22	0.04	0.04	0.02
Verbal Working Memory	WPI	0.83	0.03	0.88	0.03	0.05	0.02
	SPI	0.85	0.04	0.91	0.03	0.06	0.02
Immediate Word Memory	WPI	0.91	0.04	0.91	0.04	0.00	0.03
	SPI	0.93	0.03	0.89	0.04	−0.03	0.03
Delayed Word Memory	WPI	0.82	0.04	0.81	0.04	−0.02	0.03
	SPI	0.78	0.04	0.81	0.04	0.03	0.03

**Table 4:** Baseline scores (Mean ±SE) and percentage change observed due to treatment.

	SPI		WPI		*p*-Value ^a^
	Baseline	% Change	Baseline	% Change	
Serum B_12_ (pmol/L)	261.7 ± 8.45	↓ −9.47	260.2 ± 10.59	↓ −4.05	0.14
Active B_12_ (pmol/L)	70.16 ± 3.97	↓ −2.21	68.7 ± 4.13	↑ +19.08	<0.001
MMA (mol/L)	0.26 ± 0.021	↑ +12.24	0.26 ± 0.022	↓ −3.96	0.09
tHcy (mol/L)	11.98 ± 0.53	↑ +3.09	11.89 ± 0.55	↓ −1.78	0.14
Serum folate (nmol/L)	34.78 ± 0.26	↓ −1.05	34.45 ± 1.79	↑ +12.92	<0.01

^a^ Comparison of percentage change between treatments. ↑ Increase, ↓ decrease.

**Table 5:** Correlations between changes in biometric measures and changes in cognitive function during each treatment period; adjusted for sex and age covariates.

		Δ Serum B_12_	Δ Active B_12_	Δ MMA	Δ tHcy	Δ Serum Folate
Δ Processing Speed	WPI	0.09	0.29 *	−0.03	−0.33 *	0.22
	SPI	0.24	0.06	−0.01	0.17	0.12
Δ Reasoning Speed	WPI	−0.03	0.01	−0.11	0.21	−0.10
	SPI	0.22	0.18	−0.01	−0.49 **	0.09
Δ Numeric Working Memory	WPI	0.00	0.00	0.18	−0.31 *	0.03
	SPI	0.07	0.11	−0.11	0.03	−0.04
Δ Verbal Working Memory	WPI	0.01	0.10	0.04	−0.24	−0.10
	SPI	−0.20	−0.05	0.21	0.23	0.05
Δ Reaction Time/Attention	WPI	0.03	0.13	−0.08	−0.20	0.25 *
	SPI	−0.09	−0.03	−0.03	−0.06	−0.16
Δ Immediate Word Memory	WPI	0.11	0.07	−0.11	−0.23	0.15
	SPI	0.15	0.18	−0.02	0.24	−0.14
Δ Delayed Word Memory	WPI	0.03	−0.02	0.03	−0.06	0.39 **
	SPI	0.03	0.05	−0.15	−0.03	0.12

MMA = Methylmalonic Acid; tHcy = total homocysteine. * *p* < 0.05, ** *p* < 0.01 (one-tailed).
